# Metabolomic signature and mitochondrial dynamics outline the difference between vulnerability and resilience to chronic stress

**DOI:** 10.1038/s41398-022-01856-7

**Published:** 2022-02-28

**Authors:** Paola Brivio, Matteo Audano, Maria Teresa Gallo, Piotr Gruca, Magdalena Lason, Ewa Litwa, Fabio Fumagalli, Mariusz Papp, Nico Mitro, Francesca Calabrese

**Affiliations:** 1 Department of Pharmacological and Biomolecular Sciences, Università degli Studi di Milano, Milan, Italy; 2 Maj Institute of Pharmacology, Polish Academy of Sciences, Krakow, Poland

**Keywords:** Molecular neuroscience, Psychiatric disorders

## Abstract

Stress is the foremost environmental factor involved in the pathophysiology of major depressive disorder (MDD). However, individual differences among people are critical as some people exhibit vulnerability while other are resilient to repeated exposure to stress. Among the others, a recent theory postulates that alterations of energy metabolism might contribute to the development of psychopathologies. Here we show that the bioenergetic status in the ventral hippocampus (vHip), a brain subregion tightly involved in the regulation of MDD, defined the development of vulnerability or resilience following two weeks of chronic mild stress. Among the different metabolomic signatures observed, the glycolysis and tricarboxylic acid cycle may be specifically involved in defining vulnerability, revealing a previously unappreciated mechanism of sensitivity to stress. These findings point to mitochondrial morphology and recycling as critical in the ability to cope with stress. We show that vulnerable rats favor mitochondrial fusion to counteract the overproduction of reactive oxidative species whereas resilient rats activate fission to guarantee metabolic efficiency. Our results indicate that the modulation of the energetic metabolite profile in vHip under chronic stress exposure may represent a mechanism to explain the difference between vulnerable and resilient rats, unraveling novel and promising targets for specific therapeutic interventions.

## Introduction

Stressful life experiences result in biological and behavioral responses that increase the risk to develop stress-related pathologies, including major depression [1].

According to the concept of allostasis [2], acute stress may have positive effects, and in line we have demonstrated that acute stress improves the cognitive performance [3]. Conversely, chronic stress may induce detrimental and protracted changes [4–8] by perturbing the homeostatic network and leading to the so-called “allostatic load” and “allostatic overload”, which drive psychopathologies [9]. However, the consequences of chronic stress are not predictable. Indeed, even under chronic stress, the brain can, or cannot, activate adaptive mechanisms resulting in, respectively, resilience or vulnerability [9]. This is witnessed by the evidence that some subjects exposed to stress experience diseases while others elaborate resilience and maintain normal functions [10].

To date, despite several attempts have been made to unravel the mechanisms responsible for the development of pathological phenotypes when exposed to stress, less effort has been made to fully clarify the determinants that draw the trajectory of stress response toward resilience, i.e. the ability to adapt to adverse context [11], both at central and peripheral level [9, 12–15].

In the last few years, alterations in brain metabolism have been linked with both the pathogenesis and pathophysiology of psychiatric disorders [16]. This is corroborated by the analysis of blood and urine samples from depressed patients that show an alteration in the levels of metabolites involved in the modulation of energy and neuronal functions [17]. Consistent with this, at preclinical level, as recently reviewed by van Der Kooij [18], accumulating evidence showed metabolic alterations in animal models of psychiatric disorders based on chronic stress exposure. However, the detailed mechanisms underpinning energy metabolism in psychiatric disorders have not yet been exhaustively elucidated.

On these bases and considering the fundamental influence of bioenergetics to stress-related disorders we employed a metabolomic approach to have a more comprehensive understanding of the mechanism that may lead to susceptibility and resilience to chronic stress exposure. For this purpose, we used the chronic mild stress (CMS) paradigm, a well-characterized animal model of MDD [19], which allows stratification of this population into vulnerable and resilient groups by evaluating the hedonic phenotype [4, 5]. Indeed, anhedonia, the inability to derive pleasure from normally rewarding experiences, is one of the core symptoms of depressed patients, as listed in the DSM-5 [20]. The analyses were carried out in the ventral hippocampus (vHip) given its key role not only in the mediation of stress response and in the management of specific pathological phenotypes, including the anhedonic-like behavior [21], but also in energy metabolism [22].

Additionally, to deeper investigate the potential mechanisms underlying the metabolic changes in our experimental setting, we focused on mitochondrial dynamics whose alteration may compromise the well-being of the entire cell, thus causing the pathological conditions connected with mitochondrial homeostasis [23]. Indeed, mitochondria are the primary organelles involved in the regulation of energy production within the cell and sustain stress response system by modulating energy transformation as well as several intracellular signaling pathways [22, 24]. In this context, mitochondrial adenosine triphosphate (ATP) production is fundamental for the support of synaptic transmission and communications, the release of neurotransmitters as well as for the correct maintenance of plasticity [25, 26], all of which are essential for the proper brain functions aimed to cope with stress.

Here, we provide evidence that a peculiar mitochondrial function and energetic metabolite profile contribute to dictate the difference between resilience or vulnerable phenotype in response to stress.

## Material and methods

### Animals

Adult male Wistar rats (Charles River, Germany) were brought into the laboratory one month before the start of the experiment. Except for the first 10 days after arrival when the animals were housed in groups of 10, they were singly housed in standard laboratory conditions: except for the CMS procedure, food and water was freely available on a 12-h light/dark, constant temperature (22 ± 2 °C) and humidity (50 ± 5%). All procedures used in this study have conformed to the rules and principles of the 86/609/EEC Directive and have been approved by the Local Bioethical Committee at the Maj Institute of Pharmacology, Polish Academy of Sciences, Krakow, Poland. All efforts were made to minimize animal suffering, to reduce the number of animals used and the animal studies comply with the ARRIVE guidelines.

### Stress procedure and behavioral test

**Fig. 1:**
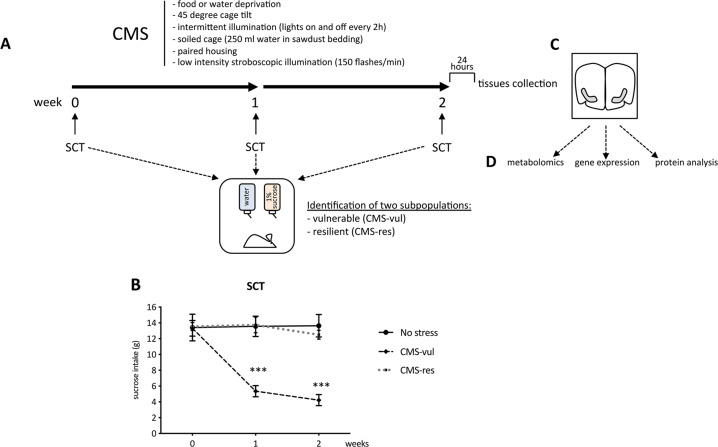
Behavioral characterization of animals exposed to chronic mild stress. **A** schematic representation of the experimental paradigm; **B** sucrose intake was measured in the sucrose consumption test (SCT) at weekly intervals in control (no stress) or stressed (CMS-vul/CMS-res) animals. The data are the mean ± SEM: ***P < 0.001 vs no stress (one-way ANOVA with repeated measures, Fisher’s PLSD); **C** schematic representation of the dissection of the vHip; **D** analysis performed in vHip.

After 2 weeks of adaptation to the housing conditions, rats were trained to consume 1% sucrose solution as previously described [5] and sucrose consumption was monitored at weekly intervals throughout the duration of the study (Fig. 1A).

On the basis of their sucrose intakes in the final baseline test, the animals were randomly divided into two matched groups: one group was subjected to the CMS for a period of 2 consecutive weeks (see: Calabrese et al. [4] for details) and the other one was not subjected to the CMS procedure (control group). On the basis of the result of the sucrose consumption test carried out following the first 2 weeks of stress, animals showing the anhedonic phenotype (CMS-vulnerable) were separated by the animals that did not develop anhedonia despite CMS (CMS-resilient) (Fig. 1B).

The animals of each experimental group were decapitated 24 h after the final sucrose test and the ventral hippocampus was dissected from the whole brain according to the plates 34–43 of the atlas of Paxinos and Watson [27] (Fig. 1C) for the subsequent molecular analysis (Fig. 1D).

Behavioral testing was done blindly by an experimenter who was unaware of the experimental group of the animals.

### Metabolomic analysis

Metabolomic data were obtained by liquid chromatography coupled to tandem mass spectrometry. We used an API-3500 triple quadrupole mass spectrometer (AB Sciex, Framingham, MA, USA) coupled with an ExionLC™ AC System (AB Sciex, Framingham, MA, USA). 10 mg of ventral hippocampus were used for the analysis. Half tissue was smashed in 250 µl of ice-cold methanol/acetonitrile 50:50, while the second half was lysed in 250 µl of ice-cold water/methanol 20:80, respectively. Both solutions contained [U-^13^C_6_]-glucose (Merck Life Science S.r.l, Milano, Italy) 1 ng/µl and [U-^13^C_5_]-glutamine (Merck Life Science S.r.l, Milano, Italy) 1 ng/µl as internal standards. Lysates were spun at 20,000×*g* for 5 min at 4 °C and supernatants were then passed through a regenerated cellulose filter (4 mm Ø). Samples were then dried under N_2_ flow at 40 °C. Samples were then resuspended in 100 µl of methanol for subsequent analysis.

Quantification of energy metabolites was performed by using a cyano-phase LUNA column (50 mm × 4.6 mm, 5 µm; Phenomenex, Torrance, CA, USA) by a 5 min run in negative ion mode with two separated runs. Protocol A: samples lysed in acetonitrile/methanol were used to analyze lactate, malate, αKetoglutarate, phosphoenolpyruvate (PEP), dihydroxyacetone-P/glyceraldehyde-3P (DHAP/GAP), erytrose-4P (E4P), dTMP, dAMP, dIMP, dCTP, ITP, and GTP. The mobile phase A was: water and phase B was: 5 mM ammonium acetate in MeOH and the gradient was 10% A and 90% B for all the analysis with a flow rate of 500 µl/min. Protocol B: samples lysed in water/methanol solution were used to analyze 3′, 5′-Cyclic GMP, acetyl-CoA, ADP, AMP, ATP, cAMP, Citrate, CMP, CoA, CTP, dADP, dATP, dCDP, dCMP, dGDP, dGMP, dGTP, dITP, dTTP, dUMP, dUTP, FAD, Fructose bis-P, Fumarate, GDP, Glucose, Glucose-6P, GMP, IMP, Iso-citrate, malonyl-CoA, NAD^+^, NADH, NADP^+^, NADPH, oxaloacetate, pyruvate, ribose-xylulose-ribulose-5P (R-X-Ru-5P), succinate, succinyl-CoA, UDP, UMP, and UTP. The mobile phase A was: water and phase B was: 5 mM ammonium acetate in MeOH and the gradient was 50% A and 50% B for all the analysis with a flow rate of 500 µl/min.

Carnitine quantification was performed on acetonitrile/methanol extracts by using a Varian Pursuit XRs Ultra 2.8 Diphenyl column. Samples were analysed by a 3 min run in positive ion mode and the mobile phase was 0.1% formic acid in MeOH.

Amino acid and biogenic amine quantification were performed through previous derivatization. Briefly, 20 µl out of 100 µl of acetonitrile/methanol samples were collected and dried under N_2_ flow at 40 °C. Dried samples were resuspended in 50 µl of phenyl-isothiocyanate (PITC), EtOH, pyridine, and water 5%:31.5%:31.5%:31.5% and then incubated for 20 min at RT, dried under N_2_ flow at 40 °C for 90 min and finally resuspended in 100 µl of 5 mM ammonium acetate in MeOH/H2O 50:50. Quantification of different amino acids was performed by using a C18 column (Biocrates, Innsbruck, Austria) maintained at 50 °C. The mobile phases for positive ion mode analysis were phase A: 0.2% formic acid in water and phase B: 0.2% formic acid in acetonitrile. The gradient was T0: 100%A, T5.5: 5%A, T7: 100%A with a flow rate of 500 µl/min. All metabolites analyzed in the described protocols were previously validated by pure standards and internal standards were used to check instrument sensitivity.

MultiQuant™ software (version 3.0.3, AB Sciex, Framingham, MA, USA) was used for data analysis and peak review of chromatograms. Raw areas were normalized by the median of all metabolite areas in the same sample. The data were then transformed by generalized log-transformation and Pareto scaled to correct for heteroscedasticity, reduce the skewness of the data, and reduce mask effects [28]. In detail, obtained values were transformed by generalized log (*glog*) as follows:

$$glog_2\left( x \right) = log_2\frac{{x + \sqrt {x^2 + a^2} }}{2}$$

where *a* is a constant with a default value of 1 and *x* is the sample area for a given metabolites [29]. Then, obtained values underwent Pareto scaling as follows:

$${{{\bar{\mathrm x}}}}_{ij} = \frac{{x_{ij} - \bar x_i}}{{\sqrt {s_i} }}$$

where *x*_*ij*_ is the transformed value in the data matrix (*i* (metabolites), *j* (samples)) and *s*_*i*_ is the standard deviation of transformed metabolite values [30]. Obtained values were considered as relative metabolite levels. Data processing and analysis were performed by MetaboAnalyst 5.0 web tool [31].

### RNA preparation and gene expression analysis by quantitative Real-time PCR

Total RNA was isolated by a single step of guanidinium isothiocyanate/phenol extraction using PureZol RNA isolation reagent (Bio-Rad Laboratories, Italy) and quantified by spectrophotometric analysis as previously described [32]. An aliquot of each sample was treated with DNase (ThermoFisher scientific, Italy) to avoid DNA contamination. Real-time polymerase chain reaction (RT-PCR) was performed to assess Catalase (*Cat*) (Rn00560930_m1, ThermoFisher scientific, Italy) mRNA levels. RNA was analysed by TaqMan qRT-PCR instrument (CFX384 real time system, Bio-Rad Laboratories, Italy) using the iScriptTM one-step RT-PCR kit for probes (Bio-Rad Laboratories, Italy) (see Brivio et al. [3] for details). Samples were run in 384 well formats in triplicate as multiplexed reactions with the normalizing internal control *36B4* (primer fw TCAGTGCCTCACTCCATCAT, primer rev AGGAAGGCCTTGACCTTTTC, probe TGGATACAAAAGGGTCCTGG). A comparative cycle threshold (Ct) method was used to calculate the relative target gene expression.

### Protein extraction and western blot analysis

Western blot analysis was used to investigate Optic Atrophy Protein 1(OPA1), mitofusin 2 (MFN2), phospho Dynamin-1-like protein (DRP1) Ser616, DRP1, oxidative phosphorylation (OXPHOS) complexes (C-I subunit NDUFB8, C-II, C-III core protein, C-IV subunit I, C-V alpha subunit), BCL2/Adenovirus E1B-interacting protein 3-like (BNIPL3/NIX), PTEN Induced Kinase 1(PINK1), PARKIN, CAT in the subcellular fractions. Tissues were manually homogenized using a glass-glass potter in a pH 7.4 cold buffer containing 0.32 M sucrose, 0.1 mM EGTA, 1 mM HEPES solution in the presence of a complete set of proteases (Roche, Monza, Italy) and phosphatase (Merck Life Science S.r.l, Milano, Italy) inhibitors. The total homogenate was centrifuged at 1000×*g* for 10 min at 4 °C to obtain a pellet enriched in nuclear components, which was suspended in a buffer (20 mM HEPES, 0.1 mM dithiothreitol (DTT), 0.1 mM EGTA) with protease and phosphatase inhibitors. The supernatant obtained was further centrifuged at 10,000×*g* for 15 min at 4 °C to obtain the pellet corresponding to the membrane fraction which was re-suspended in the same buffer prepared for the nuclear fraction. The purity of the fraction obtained was detailed in Brivio et al. [33]. Total protein content was measured according to the Bradford Protein Assay procedure (Bio-Rad Laboratories S.r.l, Segrate, Italy), using bovine serum albumin as a calibration standard. Equal amounts of protein were run under reducing conditions on 10% SDS-polyacrylamide gels and then electrophoretically transferred onto nitrocellulose membranes (Bio-Rad Laboratories S.r.l, Segrate, Italy). The blots were blocked with 5% nonfat dry milk (Euroclone, Milano, Italy) and then were incubated with the primary antibodies summarized in Table S1. Membranes were then incubated for 1 h at room temperature with the opportune secondary antibody (see Table S1). Immunocomplexes were visualized by chemiluminescence using the Western Lightning Star ECL (Euroclone, Milano, Italy) and the Chemidoc MP imaging system (Bio-Rad Laboratories S.r.l, Segrate, Italy) (see Figs. 4C, 5G, 6E, and S1C). Results were standardized using β-actin as the control protein, which was detected by evaluating the band density at 43 kDa.

### Statistical analysis

The analyses of the sucrose consumption were performed with the two-way analysis of variance (ANOVA) with repeated measures, followed by Fisher’s Protected Least Significant Difference (PLSD).

The results of the molecular analysis were analyzed with the one-way ANOVA and when appropriate, further differences were analyzed by the Fisher’s PLSD.

The metabolomics data were analyzed with the one-way ANOVA with Fisher’s PLSD with a false discovery rate (FDR) < 0.15.

Each experimental group consists of 4–8 rats. The sample size was estimated on the bases of our previous experiments. Significance for all tests was assumed for *p* < 0.05. Data are presented as means ± standard error (SEM).

## Results

### Behavioral characterization of vulnerability and resilience to chronic stress exposure

Sucrose consumption test has been employed weekly as behavioral test to discriminate the development of the anhedonic-like behavior in stressed animals (Fig. 1A). In line with our previous evidence [4, 5], two-way ANOVA with repeated measures revealed a significant effect of CMS in the 2 weeks of procedure (*F*_4-42_ = 18.03, *p* < 0.001). Indeed, 1 week of CMS induced a statistically significant reduction of the sucrose intake in a group of stressed rats (−8.1 gr, *p* < 0.001 vs no stress, Fisher’s PLSD) (Fig. 1B), named vulnerable, an effect maintained throughout the chronic stress procedure (−9.4 gr, *p* < 0.001 vs no stress, Fisher’s PLSD). By contrast, another part of stressed animals was resilient to CMS procedure, as indicated by the similar sucrose intake among non-stressed and resilient animals.

### Metabolomics signature in vulnerable and resilient animals

To test if and to what extent vulnerable and resilient phenotypes are characterized by specific metabolic patterns, we performed a targeted metabolomics to investigate nucleotide, amino acid, biogenic amine, acylcarnitine, glycolysis and tricarboxylic acid (TCA) cycle intermediate levels in ventral hippocampus of no stress, vulnerable, and resilient rats.

**Fig. 2:**
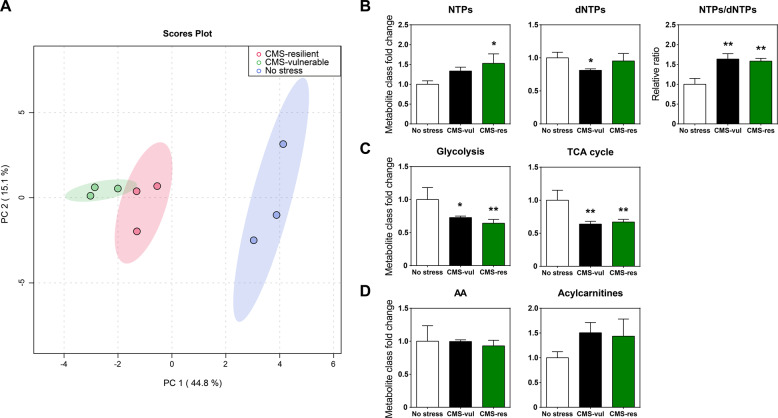
Metabolomic analysis of vHip of rats exposed to CMS. **A** principal component analysis (PCA) of targeted metabolomics; **B** fold change of nucleotides (NTPs), deoxynucleotides (dNTPs), and NTPs/dNTPs relative ratio in control (no stress) and stressed (CMS-vul/CMS-res) animals; **C** fold change of glycolysis and TCA cycle metabolites; **D** fold change of free amino acids and acylcarnitines. The data are the mean ± SEM. *p < 0.05, **p < 0.01 vs no stress; ^#^*p* < 0.05 vs CMS-vul (one-way ANOVA, Fisher’s PLSD).

Principal component analysis (PCA) unraveled a significant difference in the metabolic asset of not stressed and CMS animals, while resilient and vulnerable rats did not show any difference when compared to each other (Fig. 2A).

One-way ANOVA of the metabolic classes indicated that the sum of mono-, di- and triphosphate nucleotides (NTPs) was increased by 52.7% in resilient rats (*F*_2-6_ = 6.749, *p* < 0.05), while the sum of mono-, di-, and triphosphate dNTPs was decreased by 18.8% in vulnerable animals compared to control rats (*F*_2-6_ = 4.004, *p* = 0.0786), respectively (Fig. 2B). Noteworthy, NTPs/dNTPs ratio was increased in both vulnerable and resilient phenotypes (*F*_2-6_ = 13.58, *p* < 0.01, Fig. 2B). In addition, glycolysis (*F*_2-6_ = 8.25, *p* < 0.05) intermediates were significantly reduced by 27.2% and 35.8% in both vulnerable and resilient ventral hippocampus compared to controls, respectively (Fig. 2C). The same trend was observed for TCA cycle (*F*_2-6_ = 12.02, *p* < 0.05) intermediates, which decreased by 36% and 32.8%, respectively (Fig. 2C). No differences were detected in total levels of acylcarnitine (*F*_2-6_ = 2.584, *p* = 0.1551) and amino acid (AA, *F*_2-6_ = 0.2127, *p* = 0.8143) (Fig. 2D).

**Fig. 3:**
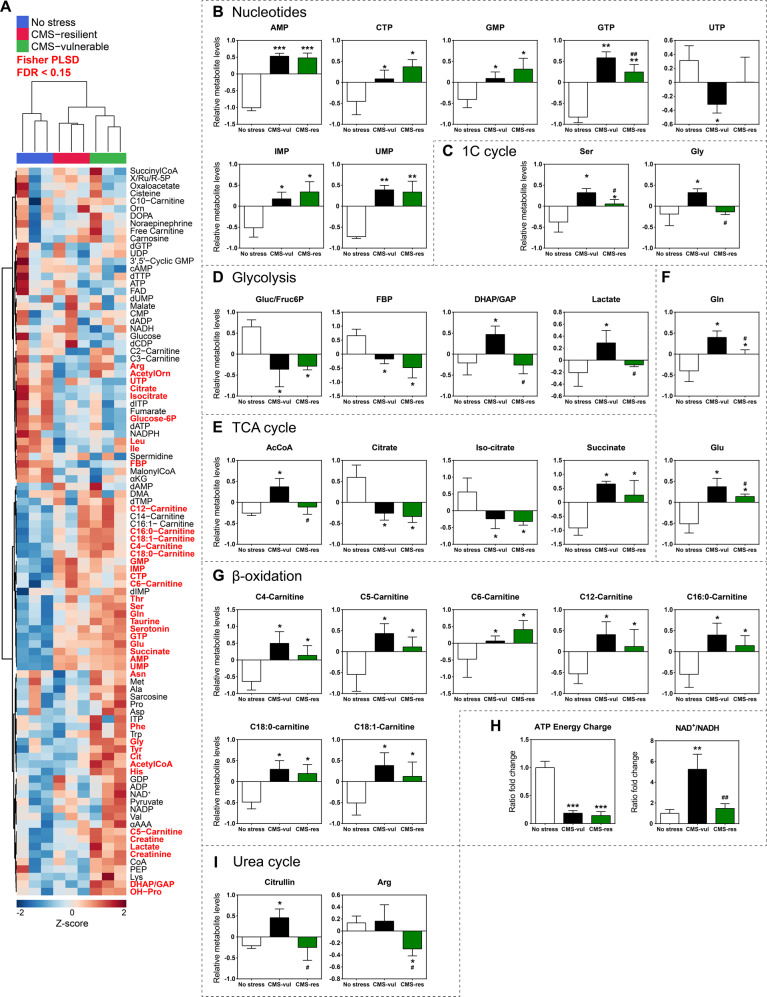
Metabolite levels in the vHip of rats exposed to CMS. **A** Heatmap representing all analyzed metabolites by targeted LC-MS/MS metabolomics. Metabolites highlighted in red were statistically significant with a FDR < 0.15; **B** relative levels of several mono-, di, and triphosphate nucleotides (NTPs), **C** relative levels of 1C cycle intermediates, **D** relative levels of glycolysis metabolites, **E** relative levels of TCA cycle intermediates, **F** relative levels of glutamine and glutamate, **G** relative levels of acylcarnitines, **H** fold change of ATP energy charge and NAD^+^/NADH ratio, panel **I**: relative levels of urea cycle intermediates in control (no stress) or stressed (CMS-vul/CMS-res) animals. **B**–**G**, **I**: the data are the mean ± SD. *FDR < 0.05, **FDR < 0.01, ***FDR < 0.001 vs no stress; ^#^FDR < 0.05, ^##^FDR < 0.01 vs CMS-vul (one-way ANOVA, Fisher’s PLSD). **H** mean ± SEM. **FDR < 0.01, **FDR < 0.001 vs no stress; ^##^FDR < 0.01 vs CMS-vul (one-way ANOVA, Fisher’s PLSD).

Prompted by these results, we investigated what metabolites were affected either by stress or vulnerable/resilient phenotype. One-way ANOVA with Fisher’s PLSD with a false discovery rate (FDR) < 0.15 identified significant variations of several metabolites levels (Fig. 3A bold red metabolites and Table S2). Several nucleotides, namely AMP, CTP, GMP, GTP, IMP, and UMP, were considerably increased in the ventral hippocampus of vulnerable and resilient rats, except for UTP (Fig. 3B). These data were associated with higher levels of one-carbon (1C) cycle serine in both vulnerable and resilient animals, and increased levels of glycine only in vulnerable rats (Fig. 3C). Notably, we did not observe changes of any specific dNTPs, despite an overall decrease in total dNTPs levels observed in vulnerable animals (Figs. 2B and 3A).

Further metabolomic data show that both vulnerable and resilient rats were characterized by significantly decreased levels of some glycolysis intermediates such as glucose/fructose-6P (Gluc/Fruc6P) and fructose-bisphosphate (FBP) compared to control rats, while only vulnerable animals showed significantly increased levels of lactate (Fig. 3D). The same trend was observed in acetyl-CoA levels, while both citrate and iso-citrate were decreased in stressed animals compared to controls (Fig. 3E). Of note, both vulnerable and resilient rats had elevated succinate as well as glutamine (Gln) and glutamate (Glu) levels, suggesting the replenishment of TCA cycle from glutaminolysis rather than β-oxidation (Fig. 3F, G). In fact, while we observed higher levels of medium (C4-, C5-, C6-, and C12-Carnitine) and long chain (C16:0-, C18:0-, and C18:1-Carnitine) acyl-carnitines levels, no differences were detected in short chain (C2- and C3-) carnitines (Fig. 3G).

The analysis of the energetic and redox state also demonstrated that stressed animals underwent a dramatic energetic depletion, even if only vulnerable rats exhibited increased levels of NAD^+^/NADH ratio (Fig. 3H). Of note, urea cycle intermediate citrulline was increased in the hippocampus of vulnerable rats, while both citrulline and arginine were remarkably decreased in resilient rats (Fig. 3I).

### Mitochondrial oxidative phosphorylation system was affected in vulnerable and resilient animals

**Fig. 4:**
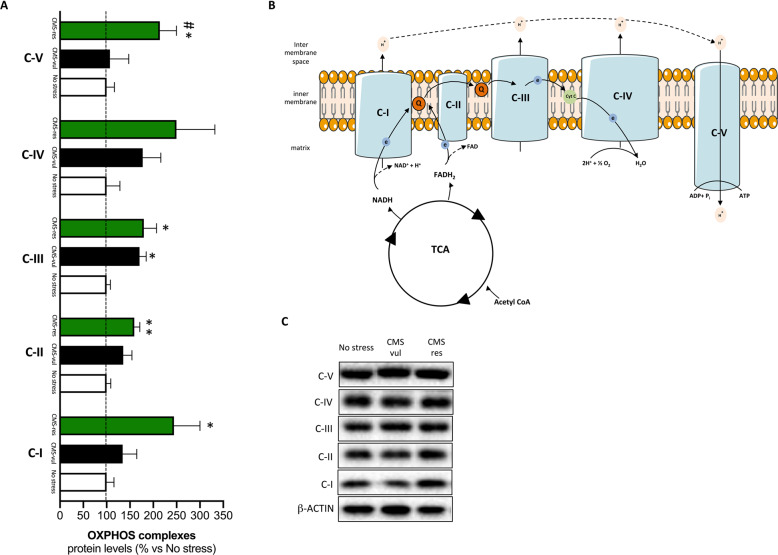
Analysis of OXPHOS complexes in the vHip of vulnerable and resilient animals to CMS. **A** OXPHOS complexes protein levels. The data are the mean ± SEM; **B** schematic representation of the electron transport chain; **C** representative WB blots. *P < 0.05, **p < 0.01 vs no stress, #*p* < 0.05 vs CMS-vul (one-way ANOVA with Fisher’s PLSD).

The differences found in several metabolic pathways and, in particular, the changes of NAD^+^/NADH ratio observed in vulnerable and resilient animals suggested a possible alteration in the mitochondrial respiratory chain. Hence, we measured the protein levels of the five multi-subunits complex of the machinery (Fig. 4B). As shown in Fig. 4A, one-way ANOVA revealed a significant effect of stress on the protein expression of C-I (*F*_2-8_ = 4.81, *p* < 0.05), C-II (*F*_2-8_ = 5.95, *p* < 0.05), C-III (*F*_2-8_ = 5.47, *p* < 0.05), and C-V (*F*_2-8_ = 4.47, *p* < 0.05). Indeed, in the PLSD post hoc analysis we found a statistically significant increase of C-I (+144%, *p* < 0.05 vs no stress), C-II (+59%, *p* < 0.01 vs no stress), and C-V (+114%, *p* < 0.05 vs no stress) protein levels specifically in resilient animals in comparison to non-stressed group. By contrast, we observed that the C-III subunit was upregulated in both CMS-vul and CMS-res animals (+71%, *p* < 0.05 vs no stress, +80%, *p* < 0.05 vs no stress respectively, Fisher’s PLSD). These results possibly indicate that the enhancement of the electron transport chain, specifically observed in resilient animals, may trigger a different mitochondrial activity to compensate the energy depletion induced by chronic stress.

### Mitochondrial dynamics and mitophagy is differently modulated by chronic stress

**Fig. 5:**
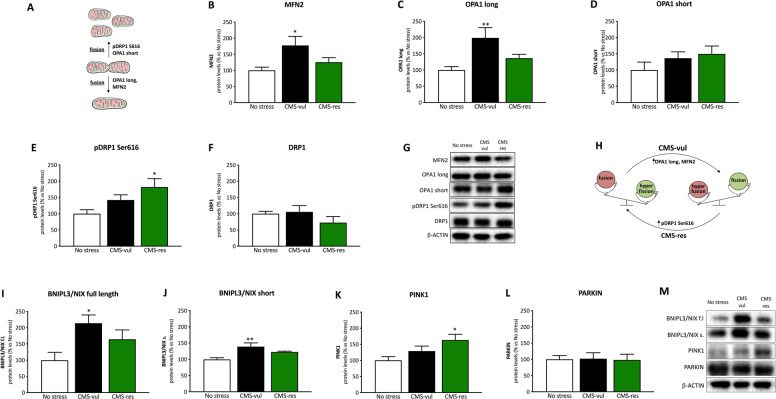
Analysis of fusion, fission and mitophagy markers in the vHip of vulnerable and resilient animals to CMS. **A** Schematic representation of the fusion and fission core machinery; Fusion (**B**–**D**), fission (**E**, **F**) and mitophagy (**I–L**) markers protein levels. The data are the mean ± SEM; **G**, **M** representative WB blots; **H**: schematic illustration of the mitochondrial dynamics unbalance. *P < 0.05, **p < 0.01 vs no stress (one-way ANOVA with Fisher’s PLSD).

Deregulation of the fusion-fission dynamics (Fig. 5A) occurs in stressful conditions [34]. Among the fusion markers we focused on MFN2 and OPA1 and we found a significant effect of stress in the one-way ANOVA (MFN2: *F*_2-9_ = 4.27, *p* < 0.05; OPA1 long isoform: *F*_2-9_ = 6.04, *p* < 0.05). Accordingly, post hoc analysis revealed a significant increase of their levels in CMS-vulnerable animals (Fig. 5B: +78%, *p* < 0.01 vs no stress; Fig. 5C: +99%, *p* < 0.01 vs no stress, Fisher’s PLSD) in comparison to the non-stressed groups, while no effect was observed in resilient rats. Moreover, the short isoform of OPA1 was not significantly modulated in these experimental conditions (Fig. 5D).

Interestingly, the increased expression of fusion markers in vulnerable animals was associated with the overexpression of Catalase mRNA (Fig. S1A: +55%, *p* < 0.001 vs no stress, Fisher’s PLSD) and protein levels (Fig. S1B: +67%, *p* < 0.05 vs no stress, Fisher’s PLSD) as highlighted by the one-way ANOVA results (*Cat*: *F*_2-21_ = 11.9, *p* < 0.001; CAT: *F*_2-9_ = 4.26, *p* < 0.05).

On the contrary, looking at the fission machinery, as shown in Fig. 5E, we observed that exposure to CMS induced an upregulation of phospoDRP1 Ser616 only in resilient animals (+82%, *p* < 0.05 vs no stress, Fisher’s PLSD) with respect to the non-stressed counterpart, as supported by the one-way ANOVA (*F*_2-9_ = 4.74, *p* < 0.05). These results suggest a different strategy of the mitochondrial dynamics in vulnerable and resilient animals to buffer the negative effects of chronic stress exposure (Fig. 5H).

In response to stressful conditions, damaged mitochondria activate mitophagy mechanisms to maintain proper cellular functions, through the modulation of mitophagy receptors [35]. Both the full length (Fig. 5I) and short (Fig. 5J) isoforms of BNIPL3/NIX, located on the outer mitochondrial membrane, were affected by stress as revealed by the one-way ANOVA analysis (*F*_2-9_ = 4.72, *p* < 0.05; *F*_2-9_ = 7.01, *p* < 0.05 respectively). Indeed, we found an upregulation of the protein levels in CMS-vul rats in comparison to non-stressed animals (full length: +114%, *p* < 0.05 vs no stress; short: +36%, *p* < 0.01 vs no stress, Fisher’s PLSD).

Another mitophagy receptor system includes the PINK/PARKIN system.

As shown in Fig. 5K, we observed a significant effect of stress in the one-way ANOVA on PINK1 (*F*_2-9_ = 4.28, *p* < 0.05), with the protein levels being upregulated specifically in resilient animals with respect to the non-stressed counterpart (+63%, *p* < 0.05 vs no stress, Fisher’s PLSD). Conversely, we did not find any changes due to CMS in PARKIN protein levels (Fig. 5L).

## Discussion

In this study, we showed that differences in the metabolic signature in the vHip characterize the vulnerability and the resilience to stress. Further, we demonstrated that while the anhedonic phenotype is linked with an increased fusion machinery, resilience to chronic stress is related to the activation of fission mechanisms to guarantee metabolic efficiency.

According to our previous evidence [4], by exposing the animals to the SCT, we were able to stratify the population of stressed animals into two different subgroups, with vulnerable animals developing the anhedonic-like behavior after 1 week of CMS while the resilient subgroup enduring resistance to the negative effects of stress that lasted throughout the whole procedure.

A critical finding of our experiments relies on the evidence that, based on PCA data, vulnerable and resilient rats are certainly different from each other but differ even more than non-stressed rats. For instance, when investigating the energetic status, vulnerable and resilient rats exhibit a profound reduction of ATP energy charge, highlighting the strong energetic susceptibility of vHip to external cues [22]; however, when we go into details and dissect the energetic status into the major classes of metabolites, i.e. glycolysis and TCA cycle, differences come up that might sustain the response to stress. In fact, among glycolysis and TCA cycle, DHAP/GAP, lactate and acetyl-CoA were all increased in vulnerable but not resilient animals, possibly indicating a different metabolism of glucose and pyruvate between vulnerable and resilient animals. Consistently, NAD^+^/NADH ratio was increased only in vulnerable rats, indicating that the conversion of pyruvate to lactate might be exploited to regenerate NAD^+^ levels in the cytoplasm. Noteworthy, altered levels of lactate were previously observed in the hippocampus of depressed rats [36]. Besides ATP production, energy metabolism provides a variety of metabolic intermediates for the generation of nucleotides, e.g. NADPH, rubose-5P, and ATP. Of note, the energetic crisis occurring in vHip of stressed animals was in line with the altered levels of nucleotides observed. In this regard, NTPs/dNTPs ratio was significantly increased in both vulnerable and resilient rats. This alteration was probably due to energetic depletion observed in the vHip of our animals, and might explain, at least in part, the dysregulation of neurogenesis observed in the vHip of several pre-clinical models of depression [12, 37–39]. 1C cycle intermediates levels support this hypothesis; indeed, serine levels were higher in vulnerable animals, while glycine was increased only in vulnerable rats.

Fatty acid β-oxidation is a major source of mitochondrial acetylCoA in many tissues. For long, the role of β-oxidation in brain homeostasis has been underestimated. Nevertheless, recent researches highlighted an important role of lipid metabolism, and more specifically of β-oxidation, in several adult brain functions [40–43]. Strikingly, we observed that many carnitines, indirect indicators of β-oxidation flux and altered in depression, were upregulated by chronic stress, independently from the behavioral phenotype [44, 45]. Specifically, we found increased levels of medium and long chain-acylcarnitines, possibly indicating an incomplete oxidation rather than an efficient fueling of carbons from fatty acids to the TCA cycle [46]. This is in line with the altered energetic profile described above. In addition, among the TCA cycle intermediates citrate and iso-citrate were the lowest abundant under stress, indicating that stressed animals are unable to efficiently convert oxaloacetate and acetylCoA to citrate. Of note, citrate synthase expression levels have been associated to cognitive decline in aged animals [47]. On the contrary, our data showed that α-ketoglutarate and other TCA cycle intermediates were unaffected or even increased, as for succinate, in the vHip of stressed animals. Together with these findings, increased levels of glutamine and glutamate in stressed animals suggest a replenishment of TCA cycle from glutaminolysis rather than from pyruvate and β-oxidation. Altered levels and transport of glutamate were also observed in previous works focused on vHip of stressed animals, suggesting a major role of this amino acid/neurotransmitter in stress adaptation [48, 49]. On the other hand, it is known that glutamine, produced by glutamine synthetase in astrocytes, is involved in the detoxification of brain ammonia [50]. This is due to the presence of incomplete urea cycle in the central nervous system, whose main function seems to be the synthesis of citrulline and arginine rather than ammonia depletion [51, 52]. Strikingly, both citrulline and arginine are involved in nitric oxide metabolism in the hippocampus, with protective effects against stress and cognitive decline [53–55]. Consistently, our data suggest a possible role of glutamine and urea cycle metabolites citrulline and arginine in the regulation of ammonia and nitric oxide levels under stress in adult resilient rats.

Accordingly, alterations of metabolites of the purine network, glycolysis, and fatty acid beta oxidation have been found in the vHip of mice exposed to the chronic social defeat animal model of depression [45], as well as changes in several metabolites in the whole hippocampus of rats subjected to the chronic mild unpredictable stress [56]. Furthermore, other authors have also shown lipidomic [56, 57] and proteomic changes [58, 59] in the brain of rodents exposed to chronic stress protocols.

Moreover, metabolic characterization of peripheral blood from MDD patients revealed disturbances of different metabolic pathways [60], including altered plasma neurotransmitter metabolite profile [61], thus increasing the knowledge about the potential molecular pathogenesis of MDD.

Prompted by the metabolic differences between vulnerable and resilient rats, we provided further support to these findings exploring the possibility that mitochondrial oxidative phosphorylation, morphology, and recycling may be part of a strategy to cope with CMS. We observed an upregulation of the protein expression of some subunits of complex I, II, and IV of the electron respiratory chain in resilient animals. In particular, the elevated levels of C-I in CMS-res underlined the unbalance in the NAD^+^/NADH and lactate levels, suggesting that the mitochondria of resilient animals have a more efficient NADH oxidation. Accordingly, recent findings demonstrated that mice with mutation of the *Ndfus4* gene, which encodes for a structural component of C-I, showed increased susceptibility to stress following 3 weeks of chronic unpredictable stress [62]. Moreover, C-II impairment has been related to ROS production and susceptibility to manifest anxiety behavior [63]. Consistently, mitochondria of vulnerable and resilient animals activate different mechanisms of morphology and recycling regulation. Vulnerable rats increase fusion machinery to cope with the excessive production of ROS, as supported by the increased levels of Cat protein and mRNA levels. In addition, vulnerable animals seem to activate the pro-apoptotic BNIPL3/NIX axis that has been found to be highly induced during hypoxia conditions and stressful conditions [64]. On the other hand, resilient animals favor mitochondrial fission likely to guarantee a higher mitochondrial quality and metabolic efficiency. In fact, the increased activation of mitochondrial fission by the pDRP1 Ser616 is associated to the PINK1-mediated mitochondrial quality control in resilient animals, potentially preventing the onset of major depression abnormalities. In line with these findings, DRP1 knockout in mouse embryonic fibroblasts showed suppressed mitophagy mediated by Parkin, while DRP1 and nitric oxide production have been recently linked to corticotrophin-releasing hormone activity in the hippocampal neurons of stressed animals [65, 66].

Taken together, these results suggest that, despite the metabolic and energetic status were profoundly affected by stress exposure independently from the behavioral phenotype, the vulnerability and resilience seem to be linked with the activation of different mitochondrial strategies set in motion to cope with negative challenges in rat vHip.

In conclusion, the modulation of the energetic metabolic profile in vHip under chronic stress exposure may represent a mechanism to explain the difference between vulnerable and resilient rats, unraveling novel and promising targets for effective therapeutic interventions.

These findings could provide novel insights into metabolic changes that could be helpful as diagnostic and predictive markers for the prevention and intervention in MDD as well as for the discovery of candidate drug targets. Indeed, the outcome of the metabolomic profile we determined in vulnerable and resilient animals may be useful in the translation “from bench to bedside” to identify innovative blood metabolite markers associated to MDD.

## Supplementary information

Supplementary materials

Supplementary Table 2

Supplementary Fig 1
