# Supplementary material for: Metabolomic signature and mitochondrial dynamics outline the difference between vulnerability and resilience to chronic stress
Source: Transl Psychiatry. 2022 Feb 28;12:87. doi: 10.1038/s41398-022-01856-7 (PMC8885712; doi:10.1038/s41398-022-01856-7)
Supplement: Supplementary file 1 — Supplementary materials [file 41398_2022_1856_MOESM1_ESM.docx]

|  | **Primary antibody** | | **Vendor/catalogue n**  **primary antibody** | | **Secondary antibody** | | **Vendor/catalogue n secondary antibody** |  |
| --- | --- | --- | --- | --- | --- | --- | --- | --- |
| pDRP1 Ser616 (78-82kDa) | 1:1000 BSA 3%, 4° O/N | Cell signaling/ 4414 | | Anti-rabbit 1:1000 BSA3%, 1h RT | | Cell signaling/7074 | | |
| DRP1 (78-82 kDa) | 1:1000 BSA 3%, 4° O/N | Cell signaling/14647 | | Anti-mouse 1:1000 BSA3%, 1h RT | | Sigma-aldrich/A4416 | | |
| OPA1 (80 kDa) | 1:1000 BSA 3%), 4° O/N | Cell signaling/80471 | | Anti-rabbit 1:1000 BSA3%, 1h RT | | Cell signaling/7074 | | |
| MFN2 (80 kDa) | 1:1000 BSA 3%, 4° O/N | Cell signaling/9482 | | Anti-rabbit 1:1000 BSA3%, 1h RT | | Cell signaling/7074 | | |
| OXPHOS (55-48-40-30-20 KDa) | 1:2000 BSA 3%, 4° O/N | Abcam/ab110413 | | Anti-mouse 1:2000 BSA3%, 1h RT | | Sigma-aldrich/ A4416 | | |
| BNIPL3L/NIX (76-38 KDa) | 1:1000 M3%, 4° O/N | Cell signaling/12396 | | Anti-rabbit 1:2000 M3%, 1h RT | | Cell signaling/7074 | | |
| PINK1 (76 KDa) | 1:1000 M3%, 4° O/N | Cell signaling/6946 | | Anti-rabbit 1:2000 M3%, 1h RT | | Cell signaling/7074 | | |
| PARKIN (52 KDa) | 1:1000 BSA 5%, 4° O/N | Genetex/GTX65811 | | Anti-rabbit 1:1000 BSA5%, 1h RT | | Cell signaling/7074 | | |
| CAT (60 KDa) | 1:1000 M3%, 4° O/N | Cell signaling/14097 | | Anti-rabbit 1:1000 M3%, 1h RT | | Cell signaling/7074 | | |
| β-ACTIN (43 kDa) | 1:10000 M3%, 4° O/N | Sigma-aldrich/A5441 | | Anti-mouse 1:10000 M3%, 1h RT | | Sigma-aldrich/ A4416 | | |

# Supplementary table 1: antibodies condition used in the western blot. BSA: Bovin serum albumin; M: Milk, O/N: overnight, RT: room temperature.

**Supplementary table 2: list of the false discovery rate (FDR) of the metabolites investigated.**

(see the excel file attached-this comment has to be removed)


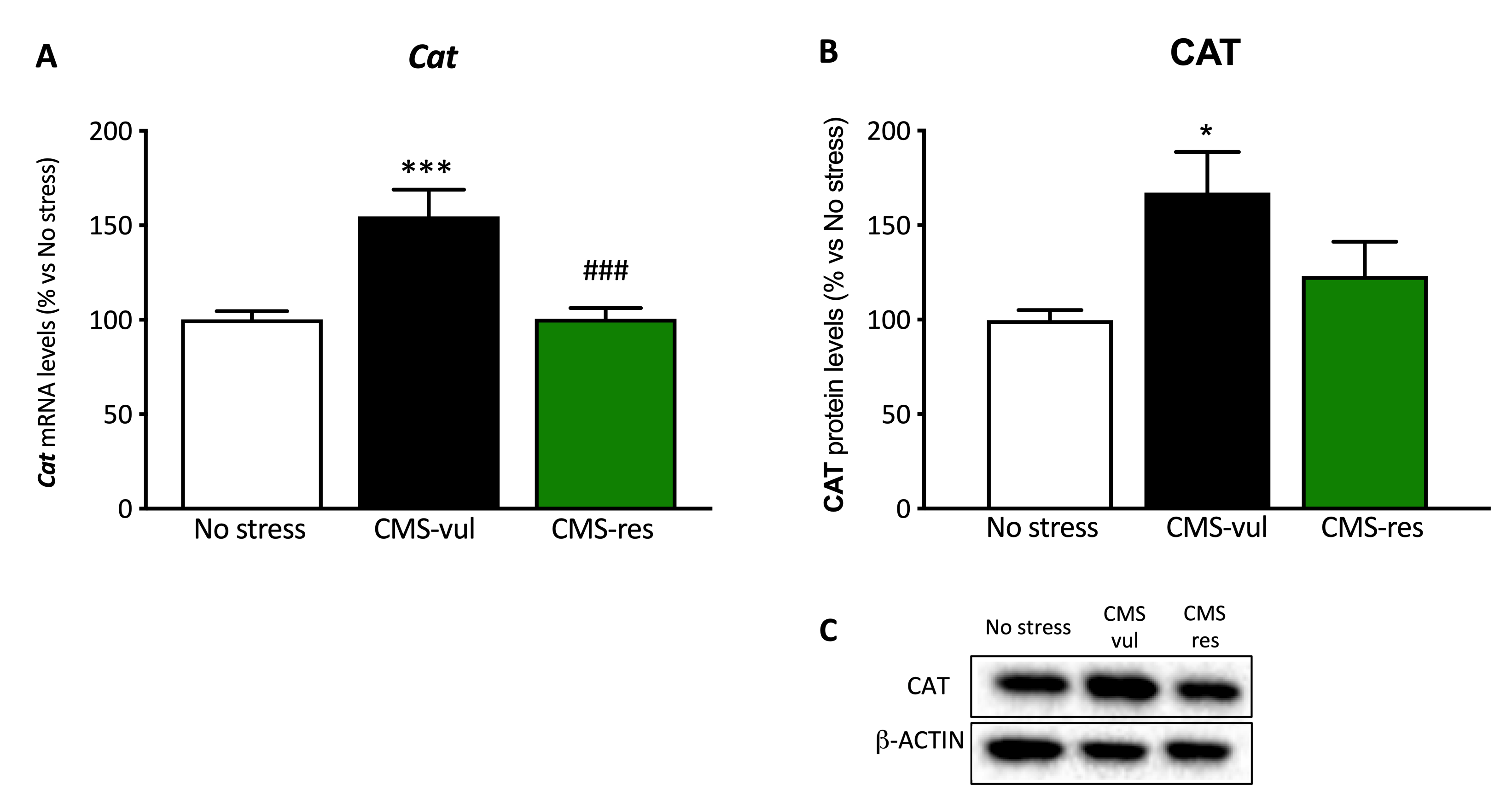


# Supplementary figure 1: Analysis of catalase mRNA (A) and protein (B) levels in the vHip of vulnerable and resilient animals to CMS. The data are the mean ± SEM. Panel C: representative WB blots. *p<0.05, ***p<0.001 vs No stress; ^###^ p<0.001 vs CMS-vul (one-way ANOVA with Fisher’s PLSD).
